# Regulation of the forming process and the set voltage distribution of unipolar resistance switching in spin-coated CoFe_2_O_4_ thin films

**DOI:** 10.1186/s11671-015-0876-5

**Published:** 2015-04-08

**Authors:** Millaty Mustaqima, Pilsun Yoo, Wei Huang, Bo Wha Lee, Chunli Liu

**Affiliations:** Department of Physics and Oxide Research Center, Hankuk University of Foreign Studies, YongIn, Gyeonggi 449-791 South Korea; Department of Physics, Semiconductor Photonics Research Center, Xiamen University, Xiamen, 361005 China

**Keywords:** Resistance switching, CoFe_2_O_4_, Set voltage, Forming process

## Abstract

We report the preparation of (111) preferentially oriented CoFe_2_O_4_ thin films on Pt(111)/TiO_2_/SiO_2_/Si substrates using a spin-coating process. The post-annealing conditions and film thickness were varied for cobalt ferrite (CFO) thin films, and Pt/CFO/Pt structures were prepared to investigate the resistance switching behaviors. Our results showed that resistance switching without a forming process is preferred to obtain less fluctuation in the set voltage, which can be regulated directly from the preparation conditions of the CFO thin films. Therefore, instead of thicker film, CFO thin films deposited by two times spin-coating with a thickness about 100 nm gave stable resistance switching with the most stable set voltage. Since the forming process and the large variation in set voltage have been considered as serious obstacles for the practical application of resistance switching for non-volatile memory devices, our results could provide meaningful insights in improving the performance of ferrite material-based resistance switching memory devices.

## Background

Resistance random access memory (RRAM), which is based on the resistance switching (RS) phenomenon in oxide thin films, has attracted much attention due to its potential applications in the next generation non-volatile memory. RRAM have been demonstrated to exhibit excellent miniaturization potential down to less than 10 nm [1], sub-ns operation speed [2,3], energy consumption (<0.1 pJ) [4,5], and high endurance (>10^12^ switching cycle) [6]. Resistance switching (RS) behavior has been reported in many oxide material-based metal/oxide/metal (MOM) structures. When the MOM structure is electrically stimulated by either a current or voltage, the resistance of the MOM structure can be switched between a high resistance state (HRS) or ‘0’ and a low resistance state (LRS) or ‘1’. Popular oxide thin films that have been studied for RRAM applications include binary oxides such as NiO [7], TaO_*x*_ [8], and HfO_2_ [9], perovskites such as Pr_1-*x*_Ca_*x*_MnO_3_ [10], La_0.7_Sr_0.3_MnO_3_ [11], and BiFeO_3_ [12]. For unipolar resistance switching (URS), the SET and RESET processes, which refer to the switching of the MOM structure from a HRS to a LRS and *vice versa*, respectively, can be induced by an applied voltage regardless of its polarity. The switching mechanism has been studied intensively, and the formation and rupture of metallic conducting filaments (CFs) have been generally accepted to explain the switching between the HRS and LRS [13].

Recently, spinel ferrites have gained interest as a potential material for RRAM applications [14-17]. Spinel ferrites have the general formula of AFe_2_O_4_, where A represents one or more divalent metal ions. Ag/ZnFe_2_O_4_/Pt structure has been reported to show RS due to the formation of CFs consisting of metallic Ag and oxygen vacancies [14]. Effort has also been paid to investigate the relation between the resistance state and the magnetic properties using Pt/NiFe_2_O_4_/Pt structures, and the results showed that the difference in the hysteresis loops at HRS and LRS was actually quite small [15]. Ag/NiFe_2_O_4_/Pt and Ag/CoFe_2_O_4_/Pt structures were reported to have RS if the films were annealed in vacuum, while no RS was found in films annealed in oxygen, indicating that oxygen vacancies played an important role [16].

Cobalt ferrite (CoFe_2_O_4_, CFO) is an important member of spinel ferrites and has gained much interest due to its rich unique magnetic and electronic properties such as magneto-optic and magneto-electric effects [17,18]. Since ferrite materials exhibit ferromagnetic behaviors but are electrically insulating, it is possible to explore both magnetic and resistance regulations in one material. Sol–gel-derived CFO and sputtering-deposited CFO thin films have been investigated for their RS properties, and the reported results mainly focused on the conduction mechanisms in the HRS and LRS [16,19]. The Pt/CFO/Pt structure has been reported to show URS with high retention capability and no detectable degradation for more than 10^4^ s [19].

In this work, we prepared CFO thin films with spin-coating and studied the effect of post-annealing conditions and film thickness on the RS properties of Pt/CFO/Pt structure. The purpose of this study is to eliminate the forming process and to reduce the set voltage (*V*_SET_) fluctuation normally observed in RRAM devices by regulating the preparation conditions of oxide thin films. For most RRAM devices, the oxide thin film is originally insulating so that a so-called ‘forming process’ is necessary to induce a soft breakdown by generating CFs in the oxide. This forming process usually occurs at a much higher voltage than *V*_SET_, which leads to high power consumption and circuit complexity. *V*_SET_ refers to the voltage value that the SET process occurs, i.e., the device is switched from a HRS to a LRS. Due to the intrinsic randomness during the formation of CFs in an insulating oxide thin film, *V*_SET_ usually has a quite wide distribution and has been considered as a serious issue to be overcome before practical applications [13]. Up to now RS behaviors in CFO thin films requiring no forming process has rarely been reported, possibly due to the quite high resistance of bulk CFO. Regarding the effect of deposition conditions on the *V*_SET_ distribution, higher annealing temperature has been shown to reduce the fluctuation of set voltages in sol–gel-prepared BiFeO_3_ [12]. Our results show that the preparation conditions of CFO thin films, i.e., post-deposition annealing temperature and film thickness, can be optimized to obtain stable RS behavior requiring no forming process, i.e., forming-free (FF) switching, and with less variation in *V*_SET_.

## Methods

The CFO thin films were prepared using a sol–gel method. Co(NO_3_)_2_ · 6H_2_O and Fe(NO_3_)_3_ · 9H_2_O were separately dissolved in 2-methoxyethanol, then the precursor solutions were mixed together with a molar ratio of Co:Fe = 1:2. The mixed solution with a total metal ion concentration of 0.2 M was then spin-coated on a Pt(111)/TiO_2_/SiO_2_/Si substrate with a rotational speed of 3,000 rpm for 30 s. After each coating, the films were dried at 170°C for 10 min and then pre-annealed at 400°C for 10 min. The procedures from coating to pre-annealing were repeated up to the desired thickness. More detailed preparation procedures have been reported previously [20]. The CFO thin films were then exposed to different heat treatment process, i.e., no further annealing (CFO-RT), post-annealed at 500°C (CFO-500) or 700°C (CFO-700) for 1 h in air ambient. To fabricate the Pt/CFO/Pt structure, 100-nm-thick Pt top electrodes were deposited on the CFO films by e-beam evaporation. The top electrode size was 90 × 90 μm.

The crystalline structure and preferential orientation of the films were characterized using the *θ* − 2*θ* scan by Rigaku X-ray diffraction (XRD) with a Cu radiation. The current–voltage (*I*-*V*) characteristics were measured using a semiconductor parameter analyzer (Agilent B1500A; Agilent Technologies, Sta. Clara, CA, USA). During the measurement, the Pt bottom electrode was grounded, and a bias voltage was applied to the top electrode.

## Results and discussion

**Figure 1:**
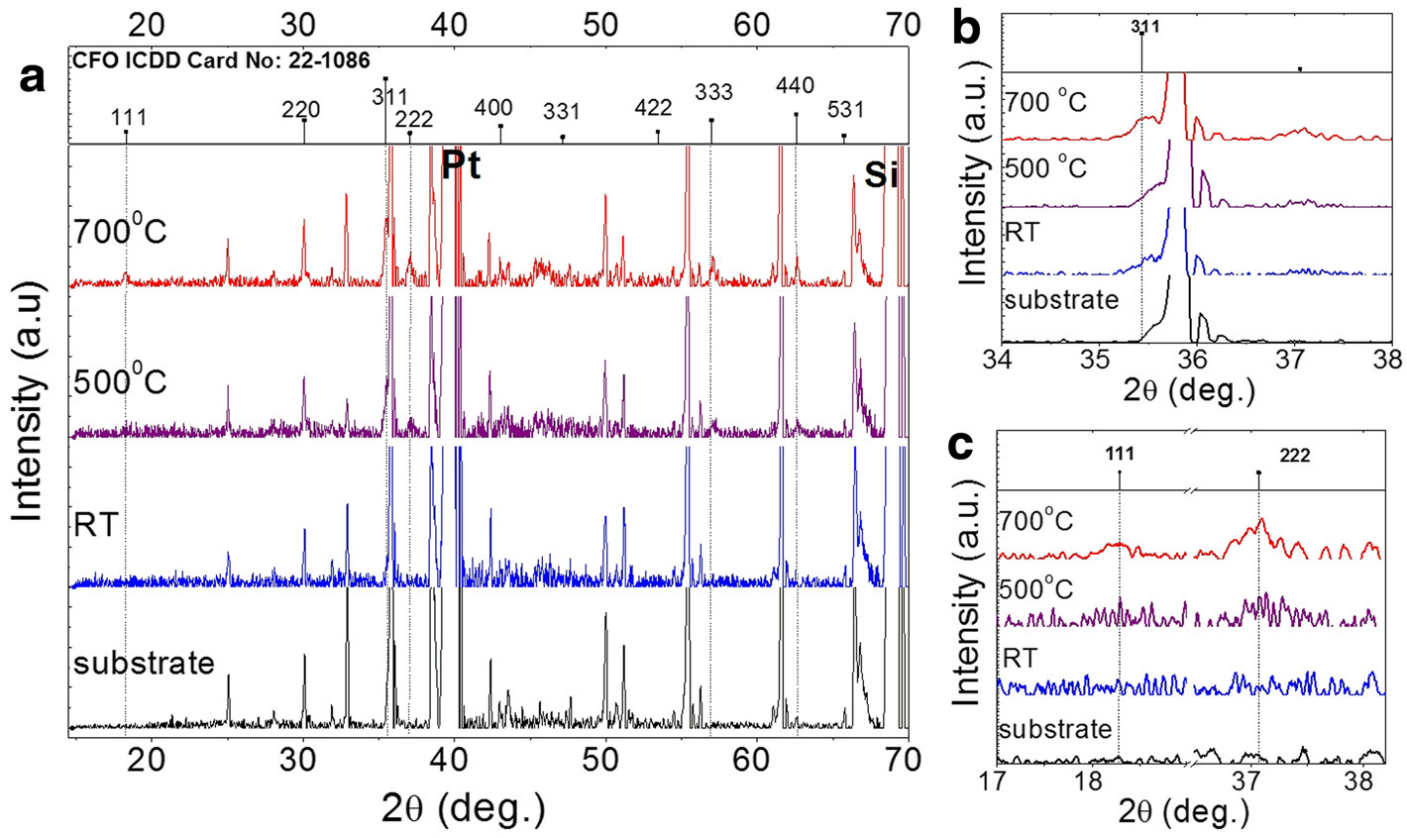
**X-ray diffraction spectra. (a)** XRD of CFO thin films with various post-annealing temperatures. **(b)** The magnified peak of (311). **(c)** The magnified peaks of (111) and (222).

Figure 1a shows the XRD patterns of the CFO with different heat treatment process. All CFO films were spin-coated three times. Weak diffraction peaks that indicate the formation of spinel CoFe_2_O_4_ started to appear in the XRD spectrum of CFO-500, and all peaks were enhanced in CFO-700. No additional peak except that from the substrate was observed from CFO-RT, indicating that the film without post-annealing was amorphous. For polycrystalline ferrite materials, the main diffraction usually appears as the (311) peak. Figure 1b showed the magnified spectra around the (311) peak, and it is clear that CFO-700 has a better crystallinity than other samples. Furthermore, we observed the (111), (222), and (333) peaks clearly in CFO-700 (Figure 1c), although these peaks normally are not seen in polycrystalline bulk CFO due to their low intensities as compared to other peaks. This preferential orientation in the (111) direction could be induced by the Pt(111) substrates. The small lattice mismatch between CoFe_2_O_4_ and Pt induced the nucleation of CFO grains with preferred (111) orientation on top of the (111)-oriented Pt substrate [21].

**Figure 2:**
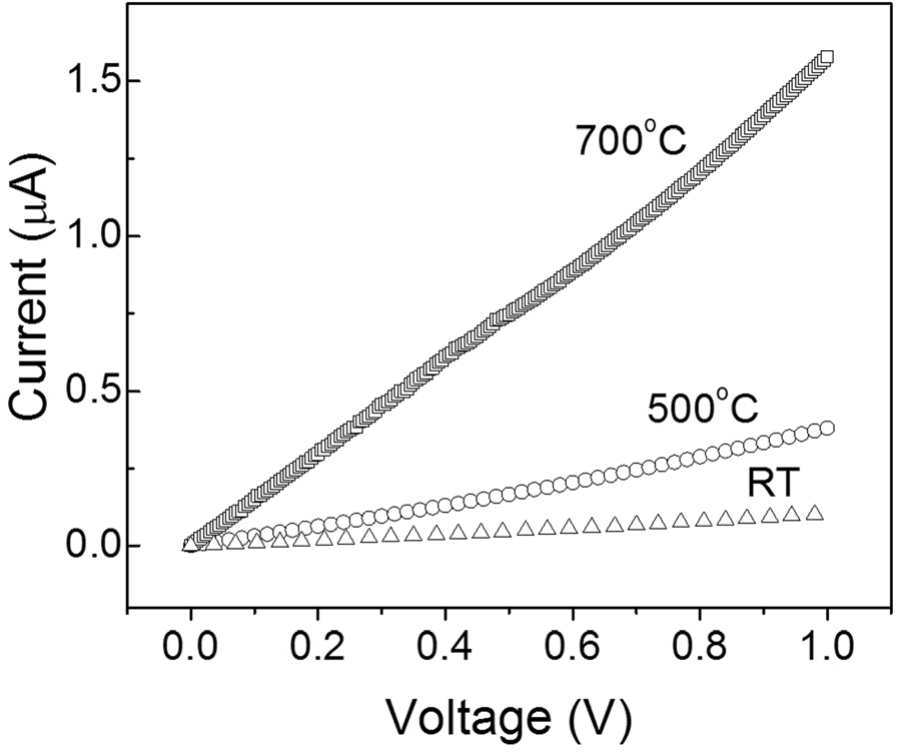
**Initial resistance.** *I*-*V* characteristics of pristine CFO films grown at different post-annealing temperatures.

Figure 2 depicts the typical *I*-*V* characteristics of the as-fabricated Pt/CFO/Pt structures measured at low bias voltages. Based on the change in the slope of the *I*-*V* curve, it is clear that the initial resistance of the device has been significantly affected by the post-deposition heat treatment of the CFO thin films. The initial resistance of the Pt/CFO-RT/Pt structure is about 10 MΩ, and this value decreased to approximately 4 MΩ for Pt/CFO-500/Pt and 2 MΩ for Pt/CFO-700/Pt. Furthermore, about 17% of Pt/ CFO-700/Pt showed even smaller resistance approximately 50 Ω (data not shown).

**Figure 3:**
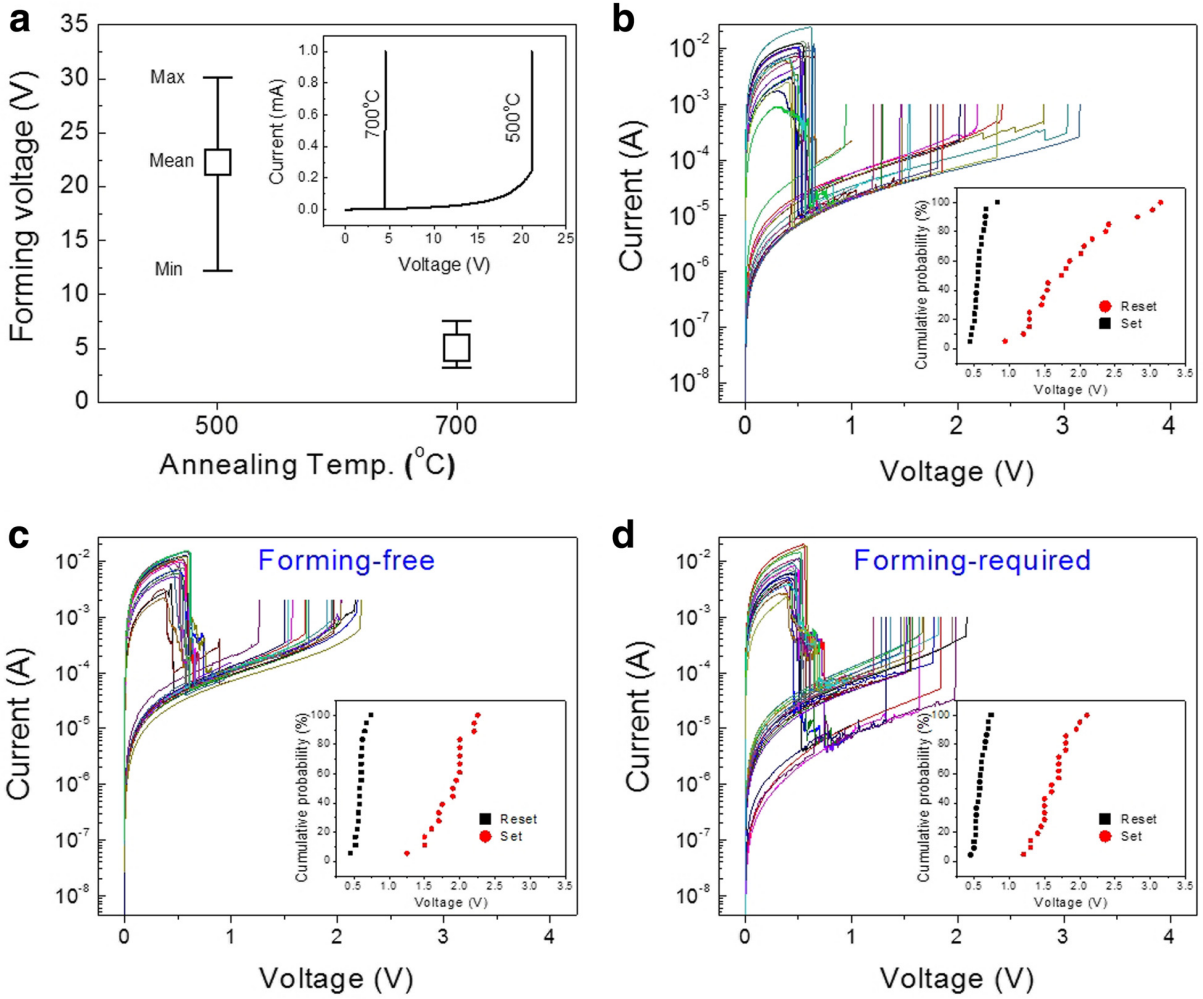
*I* **-** *V* **characteristics. (a)** Post-annealing temperature effect on the forming voltage. The inset shows the forming process. **(b)** Typical *I*-*V* of CFO-500. **(c)** Typical *I*-*V* of CFO-700 without forming process. **(d**) Typical *I*-*V* of CFO-700 with forming process. The insets in (b) to (d) are the distribution of the set and reset voltages.

The RS properties of the Pt/CFO/Pt structures were directly affected by their initial resistance. Pt/CFO-RT/Pt did not show resistive switching, presumably due to the very high initial resistance to initiate soft breakdown inside the film. URS was observed from both Pt/CFO-500/Pt and Pt/CFO-700/Pt, but the stability and reproducibility of RS were much better in Pt/CFO-700/Pt. Although the Pt/CFO-500/Pt structures showed unipolar RS, most devices could no longer be switched after just a couple of switching cycles. Additionally, the forming voltage of Pt/CFO-500/Pt was much larger as compared to Pt/CFO-700/Pt as shown in Figure 3a. The average forming voltage of Pt/CFO-500/Pt was around 20 V with a wide distribution, whereas the forming voltage of most Pt/CFO-700/Pt was about 5 V. The lower initial resistance and forming voltage observed in Pt/CFO-700/Pt could be a result of increased oxygen vacancies and less grain boundaries. In addition to the improved crystallinity evidenced by the XRD result (Figure 1), a higher growth temperature or annealing temperature has been reported to induce a higher oxygen vacancy concentration in oxide thin films prepared by pulsed laser deposition or sol–gel [12,22]. The oxygen vacancies are known to form and migrate predominantly along the grain boundaries [23]; therefore, the existence of more oxygen vacancies and less randomly distributed grain boundaries seems to facilitate the formation of CFs, resulting in a lower forming voltage in CFO-700.

The RS *I*-*V* curves of one Pt/CFO-500/Pt device which showed repeated switching are shown in Figure 3b. The reset voltages (*V*_RESET_) are quite uniform (0.5 ~ 0.75 V), whereas the range of the *V*_SET_ are much broader (1 ~ 3.2 V) as seen in the inset of Figure 3b. For Pt/CFO-700/Pt, two types of RS behaviors regarding the forming process were observed. The devices which have low initial resistance approximately 50 Ω can be switched directly from LRS to HRS; therefore, no forming process was necessary. This FF URS switching *I*-*V* characteristics is shown in Figure 3c. Those devices with higher initial resistance around MΩ, however, required a forming process to initiate RS. The typical forming-required (FR) URS *I*-*V* curves are shown in Figure 3d. Besides the difference in forming process, a comparison between the set voltages of Pt/CFO-500/Pt and Pt/CFO-700/Pt (inset of Figure 3b, c, and d) clearly shows that the distribution of the *V*_SET_ is much smaller in Pt/CFO-700/Pt. Therefore, considering the forming process and the switching stability, we chose Pt/CFO-700/Pt for further investigation on the RS properties.

**Figure 4:**
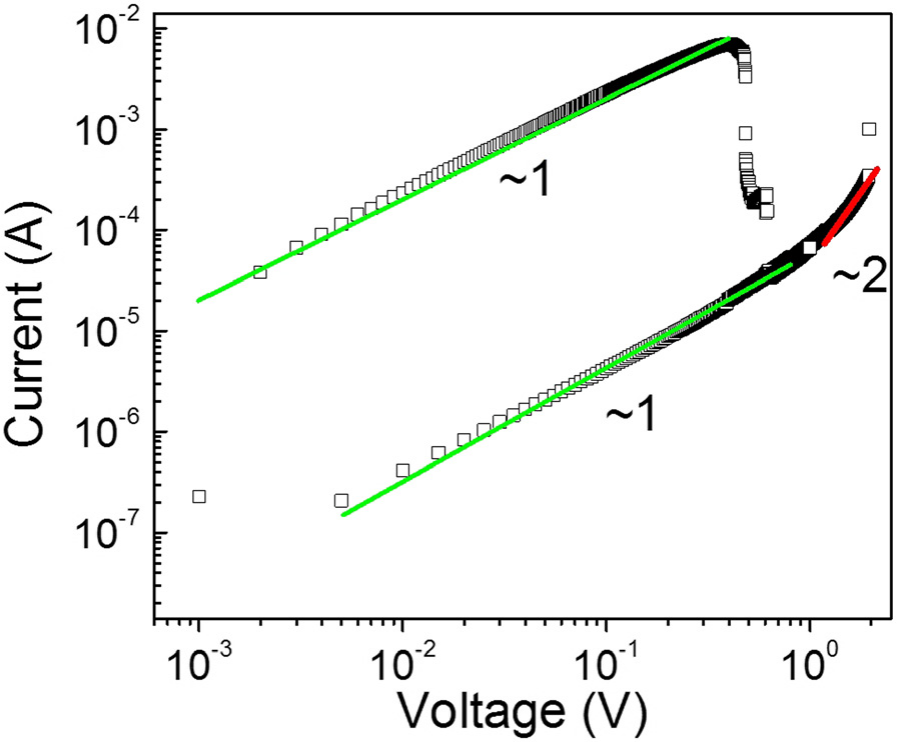
**Conduction mechanisms**. The logarithmic plot and linear fitting of *I*-*V* data of CFO-700.

To understand the conduction mechanisms of CFO thin films, the *I*-*V* curves of the Pt/CFO-700/Pt devices at HRS and LRS were plotted in a log-log scale as shown in Figure 4. In the LRS, *I*-*V* curves exhibited a linear dependence on voltage with a slope of nearly 1, which satisfies the ohmic behavior. This is thought to correspond to the formation of metallic CFs during the SET process. However, the conduction mechanism of HRS is more complicated. The fitting results suggested that the leakage current at the lower voltage region showed a linear dependence on voltage, whereas at voltages higher than 0.7 V, the current exhibited a dependence on the square of voltage. Accordingly, the conduction mechanisms of HRS can be explained as follows. For the bias voltage less than 0.7 V, thermally generated charge carriers dominated the conduction and followed the ohmic law. When the bias voltage increased, injected carriers started to increase. However, oxygen vacancies or other defects existed in CFO could behave as traps and capture the injected carriers [16,19]. The square law shown for voltages higher than 0.7 V implied that under this condition, the traps in the CFO thin films were almost filled and the *I*-*V* characteristics were governed by a trap-free space-charge limited (SCL) conduction [24]. Similarly, RS behaviors in ZnFe_2_O_4_ and other reported CoFe_2_O_4_ thin films have also been suggested to follow the SCL conduction at high field in the HRS [14,16,25].

In addition to the heat treatment effect, we further studied the dependence of RS behavior on the thickness of the CFO thin film. From our previous work [20], the thickness of the CFO films post-annealed at 700°C was about 50 to 60 nm for one-time spin-coating, and the CFO thin film thickness increased almost linearly with the number of coating layers. In this work, the thickness of the CFO films was varied by adjusting the number of spin-coated layers from 1 to 4, and the CFO films were labeled as CFO-1, CFO-2, CFO-3, and CFO-4 accordingly.

**Figure 5:**
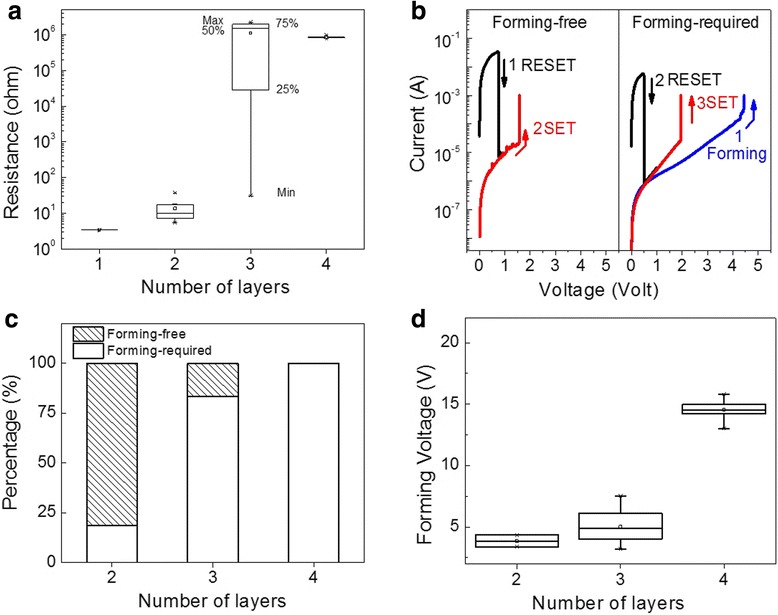
**Statistical analysis of** ***I-V*** **data of CFO films for various layers. (a)** Initial resistance, **(b)** typical *I*-*V* of forming-free and FR RS, **(c)** the percentage of forming-free and forming-required devices, and **(d**) the forming voltage distribution.

The initial resistance of CFO with various layers is displayed in Figure 5a. With increasing thickness, the initial resistance increased from the order of 10^0^ Ω for CFO-1 up to the order of 10^6^ Ω for CFO-4. Accordingly, different RS behaviors were observed from the CFO thin film depending on their thickness. CFO-1 did not show RS due to its much lower resistance that could not be switched to a HRS. From CFO-2 to CFO-3, both FF and FR RS were observed, and the typical switching characteristics are shown in Figure 5b. Due to the range of the initial resistance in CFO-2 and CFO-3, the devices with low resistance exhibited the FF switching, whereas the devices of high initial resistance started the FR switching with a forming process. Interestingly, the percentage of the FF and FR devices changed with the CFO film thickness, as shown in Figure 5c. Nearly 80% of the Pt/CFO-2/Pt devices showed FF switching, but the percentage decreased to about 20% in the Pt/CFO-3/Pt devices. This result is in a good agreement with the distribution of the initial resistance illustrated in Figure 5a, considering the relatively lower initial resistance of Pt/CFO-2/Pt and the wide-ranged initial resistance of Pt/CFO-3/Pt overlapped with Pt/CFO-2/Pt. When the CFO thin film was spin-coated four times, all devices showed FR switching, which can be attributed to the higher initial resistance due to increased film thickness. For all FR devices with different CFO film thicknesses, we observed that the forming voltage showed an increasing tendency with the film thickness (Figure 5d), which is also consistent with the results shown in Figure 5a.

**Figure 6:**
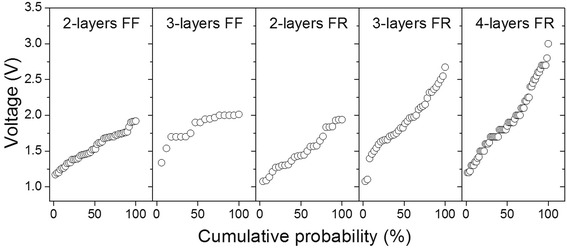
**The distribution of set voltages vs. layer numbers and forming behaviors.**

The diversity in the forming process and the CFO film thickness were found to have a significant effect on the distribution of the *V*_SET_, which has been considered as an important parameter in evaluating the performance of an RRAM device. The *V*_SET_ distribution dependence on the CFO film thickness and the forming behavior were plotted in Figure 6. The data taken in Figure 6 were taken from multidevices on each film. Regarding the CFO film thickness, it is obvious that the *V*_SET_ has a much wider distribution in Pt/CFO-4/Pt devices. Furthermore, the FF devices of Pt/CFO-3/Pt structures showed a significant narrowing in the range of the *V*_SET_ as compared to the FR devices. The FF devices usually have a low initial resistance due to the large amount of pre-existed CFs in the oxide thin films [23]. The FF RS starts with a RESET process that switches the device from the LRS to the HRS, rupturing some of the pre-existed CFs. During the SET process which switches the device from HRS back to the LRS, the CFs are regenerated preferentially along similar paths; therefore, the *V*_SET_ shows less fluctuation. On the other hand, the FR devices usually require higher and wider ranged set voltages to reconnect the CFs due to the insulating nature of the oxide film [8]. In this point of view, it is preferred to use Pt/CFO-2/Pt structures for RRAM applications because most of the CFO-2 based devices are FF with a narrowly distributed *V*_SET_, as shown in the first two panels of Figure 6. In this case, reduced power consumption and better operation stability can be expected due to the elimination of the forming process and less variation in the *V*_SET_.

## Conclusions

We have prepared CFO thin films on Pt/TiO_2_/SiO_2_/Si substrates using spin-coating with various post-annealing conditions and film thickness. Pt/CFO/Pt structures were fabricated using these CFO thin films to investigate the RS properties. Reproducible URS was observed only from CFO thin films post-annealed at 700°C, and the LRS and HRS can be explained with the ohmic conduction and SCL conduction mechanisms, respectively. The thickness dependence investigation revealed that RS started to appear from the Pt/CFO-2/Pt structures and was dominated by the FF switching. With increasing CFO film thickness, FR switching became dominant. Due to the large amount of pre-existed conducting filamentary paths in the FF RS devices, the *V*_SET_ was found to be more stable than in the FR RS devices; therefore, the Pt/CFO-2/Pt structure was preferred for potential practical RRAM applications.

## Abbreviations

CF: conducting filament
CFO: cobalt ferrite
FF: forming-free
FR: forming-required
HRS: high resistance state
LRS: low resistance state
RRAM: resistance random access memory
RS: resistance switching
SCL: space-charge limited
XRD: X-ray diffraction
